# Miscellaneous

**Published:** 1891-08

**Authors:** 


### MISCELLANEOUS.

### DRIVER ANTS.

There are certain ants that show wonderful intelligence, and the "driver ants" not only build boats, but launch them, too; only these boats are formed of their own bodies. They are called "drivers" because of their ferocity. Nothing can stand before the attacks of these little creatures. Large pythons have been killed by them in a single night, while chickens, lizards, and other animals in Western Africa, flee from them in terror. To protect themselves from the heat, they erect arches under which numerous armies of them pass in safety. Sometimes the arch is made of grass and earth gummed together by some secretion 'and again it is formed by the bodies of the larger ants, which hold themselves together by their strong nippers, while the workers pass under them. At certain times of the year, freshets overflow the country inhabited by the "drivers," and it is then that these ants go to sea. The rain comes suddenly, and the walls of their houses are broken in by the flood, but instead of coming to the surface in scattered hundreds, and being swept off to destruction, out of the ruins rises a black ball that rides safely on the water and drifts away. At the first warning of danger, the little creatures run together, and form a solid ball of ants, the weaker in the center ; often this ball is larger than a common base-ball and in this way they float about until they lodge against some tree, upon the branches of which they are soon safe and sound.—*St. Nicholas.*

### FORMATION OF CHALK.

A French scientist, M. Walther, has made some observations in the Mediterranean Sea of the manner in which chalk is formed by sea weeds. He particularly studied the *Lithotamnia* of the Bay of Naples, which grow at depths of from one hundred to three hundred feet, a class of algæ remarkably poor in organic matter, but rich in mineral constituents, among which carbonate of lime is preponderant. They grow to be about as large as the hand, and then die without suffering change of form by decomposition. Living plants attach themselves to dead ones, and thus extensive deposits are formed. Beds of pure, uncrystalized chalk remain after the gradual disappearance of the organic matter, the vacancies left by which are gradually filled with calcareous substance. Beds of chalk thus formed may, under some conditions, attain great thickness.

### HOW TO KEEP FLIES FROM PICTURE FRAMES.

Boil three or four onions in a pint of water; then with a gilding brush go over the frames of your pictures and chimney glasses, and rest assured that the flies will not alight on the articles washed with the solution. It will do no injury to the frames.

### ECONOMY.

As a rule, "economy" must be the watchword in every branch of home management, if comfort and independence are to be attained. But there are people who can only be economical on a large scale. They keep a sharp eye on the sovereigns and half sovereigns, but the small sums trickle through their fingers unobserved. They are adepts at driving a bargain, as many a poor tradesman knows to his sorrow, and that is their highest notion of thrift. The weakness lies in not being able to get the full value out of the article purchased. Wearing apparel is thrown aside as being worn out, when a little scheming and restoring would render it serviceable for months to come; food is carried from the table and condemned as waste material for want of a little skill and patience in re-cooking and daintily serving it; and odds and ends of every description, which might easily be dispensed with, are bought for no particular reason but to satisfy the desire for possession. Some of my readers who have never known the pinch of a small income may not see the need of economy in their circumstances; but the fact that we have plenty of money to-day is no proof that want will never come. The most promising of prospects are often blighted, and the happiest homes broken up, and people who have been reared in the lap of luxury have felt, in after years, the necessity of retrenchment in household management. The best preparation for such an unwelcome emergency is a habitual carefulness about the little incomes and expenditures, which, though trifling, perhaps, in themselves, make a serious difference in the home finances in the course of a year.

### QUEEN VICTORIA'S DINING ROOM.

The Queen's dining room always presents a very bright, brisk aspect, for in addition to the crowd of servants in their royal liveries, and some of her majesty's Highland and Indian domestics, there are head functionaries of the kitchen and clerks of the cellar in their respective uniforms. The clerk of the kitchen, who is at the head of the cuisine department, receives £700 a year, with board and lodging, and is provided with a staff of four assistant clerks and a female menial, who is officially known as the "necessary woman." The chief is aided by four master cooks, two yeomen of the kitchen, one of whom is a confectioner, two bakers, two roasting cooks, two coffee women, and a perfect regiment of assistants, male and female, apprentices, scourers, kitchen maids, two steam apparatus workers, two green office men, a storekeeper. The cellar is a different department.

### SPIRITUAL.

Michael Conley died in Dubuque, Ia., a short time ago. His body was taken to the Morgue and the clothes he had on were thrown aside.

When his daughter in Chickasaw County heard of his death she fell into a swoon. She dreamed she saw the clothes he wore when dying and received from him a message, saying that he had sewed up a roll of bills in his shirt. On recovering consciousness she demanded that some one go to Dubuque and get the clothes. In order to quiet her mind her brother visited that city, received the clothes from the coroner and found the money sewed in the shirt with a piece of his sister's red dress, exactly as she had described, though she had known nothing about the patch or the money.—*N. Y. World.*

An Open Competitive Examination of candidates for Junior Assistant Physician in the State Hospitals and Asylums, will be held at the office of the Civil Service Commission, Albany, N. Y., Thursday, August 20, 1891, commencing at ten o'clock, A. M. A candidate for the position must be a citizen of the State of New York, at least 21 years of age, and have had at least one year's experience in a hospital, or three years' experience in the general practice of medicine. For application blank address the Secretary of the New York Civil Service Commission, Albany, N. Y.

EDITOR HALL'S JOURNAL OF HEALTH.

Sir—Some four months ago I wrote you respecting the Herba Vita, advertised in your publication. I stated that I was afflicted with a disease of the skin. You did not claim to know as much about Herba Vita as I thought you ought to, nevertheless, I sent for it and found to my great relief it was just what I wanted. I had tried many external remedies without any benefit, but by following the directions and taking Herba Vita regularly for six or seven weeks, the skin difficulty has entirely disappeared and my general health is better than it has been for some years.

MRS. CLARISSA DRUMMOND.

Circleville, June 29, 1891.

FOR DYSPEPSIA, USE HORSFORD'S ACID PHOSPHATE.—Dr. J. J. McWilliams, Denison, Ia., says: "I have used it largely in nervousness and dyspepsia, and I consider that it stands unrivalled as a remedy in cases of this kind. I have also used it in cases of sleeplessness, with very gratifying results."

